# Morphological MRI phenotypes of multiple sclerosis differ in resting-state brain function

**DOI:** 10.1038/s41598-019-52757-7

**Published:** 2019-11-07

**Authors:** Daniela Pinter, Christian F. Beckmann, Franz Fazekas, Michael Khalil, Alexander Pichler, Thomas Gattringer, Stefan Ropele, Siegrid Fuchs, Christian Enzinger

**Affiliations:** 1 Department of Neurology, Medical University of Graz, Auenbruggerplatz 22, Graz, Austria; 2 Research Unit for Neuronal Plasticity and Repair, Medical University of Graz, Auenbruggerplatz 22, Graz, Austria; 3 Donders Institute, Cognitive Neuroscience Department and Centre for Cognitive Neuroimaging, Radboud University Nijmegen, Kapittelweg 29, Nijmegen, The Netherlands; 4 Division of Neuroradiology, Vascular and Interventional Radiology, Department of Radiology, Medical University of Graz, Auenbruggerplatz 9, Graz, Austria

**Keywords:** Neuroscience, Multiple sclerosis

## Abstract

We aimed to assess differences in resting-state functional connectivity (FC) between distinct morphological MRI-phenotypes in multiple sclerosis (MS). Out of 180 MS patients, we identified those with high T2-hyperintense lesion load (T2-LL) and high normalized brain volume (NBV; a predominately white matter damage group, WMD; N = 37) and patients with low T2-LL and low NBV (N = 37; a predominately grey matter damage group; GMD). Independent component analysis of resting-state fMRI was used to test for differences in the sensorimotor network (SMN) between MS MRI-phenotypes and compared to 37 age-matched healthy controls (HC). The two MS groups did not differ regarding EDSS scores, disease duration and distribution of clinical phenotypes. WMD compared to GMD patients showed increased FC in all sub-units of the SMN (sex- and age-corrected). WMD patients had increased FC compared to HC and GMD patients in the central SMN (leg area). Only in the WMD group, higher EDSS scores and T2-LL correlated with decreased connectivity in SMN sub-units. MS patients with distinct morphological MRI-phenotypes also differ in brain function. The amount of focal white matter pathology but not global brain atrophy affects connectivity in the central SMN (leg area) of the SMN, consistent with the notion of a disconnection syndrome.

## Introduction

Multiple sclerosis (MS) is characterized by an inflammatory and neurodegenerative component and represents the major cause for non-traumatic disability in young adults^1^. While the complex pathogenesis of MS is incompletely understood, previous studies showed that inflammatory activity and neurodegeneration may to some degree be independent of each other^2–5^.

MRI is the best-studied tool to indirectly depict pathophysiologic mechanisms in MS *in vivo*^2^. Considering the heterogeneity of MRI findings even between patients with the same clinical phenotype, a categorization of patients based on morphological MRI characteristics has been proposed^2,3,6–8^.

While many patients display congruent extremes concerning the degree of focal inflammation and diffuse neurodegeneration, some MRI studies suggested that approximately 25–50% in fact may demonstrate a dissociation between inflammatory (predominantely white matter damage) and neurodegenerative (predominantely grey matter damage) pathology^2,3,8^.

Moreover, physical deficits in MS cannot fully be explained by cerebral structural damage, but also correlate with functional imbalances in and between brain networks^9^. Functional MRI (fMRI) studies have provided important insights into the role of functional reorganization related to structural damage in MS^9–11^. Studying functional connectivity (FC) using resting-state fMRI (rfMRI) is particularly attractive, as it remains uninfluenced by task performance^12^.

Previous studies showed increased physical disability in MS to be related to decreased FC in the default-mode network (DMN)^13,14^. In contrast, correlations between FC changes in the sensorimotor network (SMN) and physical disability were not consistently observed^11,15–18^. One possible explanation for these inconsistent correlations might be FC variations between MRI-phenotypes. We here aimed to assess potential differences in resting-state FC between MS patients with predominately white matter damage versus grey matter damage MRI-phenotypes.

## Methods

### Participants

This study is an analysis of a prospective study of patients with MS and healthy controls (HC) investigated between 2014 and 2017. The current study sample was selected according to the MRI morphologic criteria described below from a cohort of 180 MS patients^19^ from our outpatient MS clinic who had consecutively undergone brain MRI over a period of 26 months at the same 3 T scanner applying identical protocols including rfMRI data acquisition.

Patients had to have no relapse within the previous two months, had not received corticosteroids eight weeks prior to scanning, and had no history of serious psychiatric illness (e.g. depression) or other neurologic disorders than MS. Disability was assessed using the Expanded Disability Status Scale (EDSS) at the time of MRI scanning.

We also assessed 37 age-matched healthy controls (HC) free of neurological diseases, using the same protocol at the same scanner.

### Standard protocol approvals, registrations, and patient consents

The study was approved by the ethics committee of the Medical University of Graz. The study was carried out in accordance with relevant guidelines and regulations. All participants gave written informed consent.

### MRI data acquisition

MRI was performed on a 3 Tesla TimTrio scanner (Siemens Healthcare, Erlangen, Germany) using a 12-channel head coil. High-resolution structural 3D images were acquired by means of a T1-weighted MPRAGE sequence with 1 mm isotropic resolution (TI = 900 ms, TR = 1900 ms, TE = 2.19 ms, 176 slices). A T2-weighted fluid-attenuated inversion recovery (FLAIR) sequence with 1 × 1 × 3 mm³ resolution served for the assessment of the hyperintense T2-lesion load (T2-LL) in the patients (TI = 2500 ms, TR = 9000 ms; TE = 69 ms, 44 slices). rfMRI data were acquired with a single shot echo planar imaging sequence with 3 × 3 × 3 mm resolution (TR = 3000 ms; TE = 30; 150 volumes, field of view = 192 × 192 mm², Matrix = 64 × 64, 36 slices, slice thickness = 3 mm, acquisition time = 7.5 minutes). Participants were asked to close their eyes during rfMRI. The total imaging time was approximately 20 minutes.

### Structural MRI analyses

Given our focus to investigate potential differences in SMN FC using a clinically applicable subtyping based on conventional MRI sequences, we chose to estimate the amount of grey matter damage on T1-weighted MPRAGE sequence and the amount of white matter damage on FLAIR images. The extent of grey matter damage was estimated by the normalized brain volume (NBV; in cm³) and the burden of focal white matter damage was estimated by the T2-lesion load (T2-LL)^4^.

After lesion filling with the FSL lesion filling toolbox, NBV was assessed from the T1-weighted MPRAGE images using SIENAX (Structural Image Evaluation, using Normalization, Single-Time-Point Estimation v 2.6), part of the FMRIB Software Library (FSL)^20^. T2-LL was assessed by a semi-automated region growing algorithm^21^ subsequent to lesion identification by a single experienced rater (CE).

**Figure 1:**
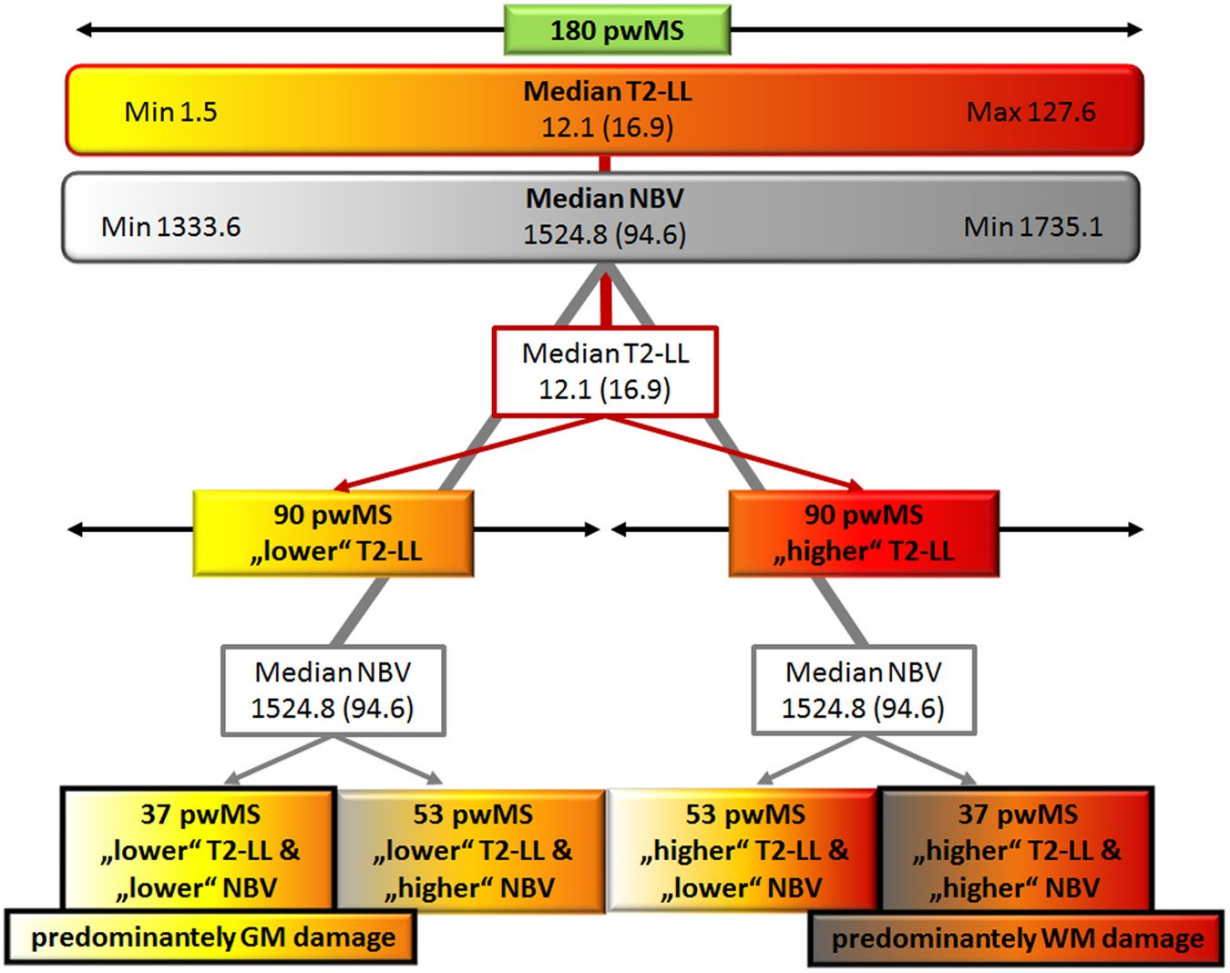
Overview of stratification procedure classifying patients with higher white vs. grey matter damage by median-split.

We applied a median-split for NBV and T2-LL to identify distinct morphological phenotypes with a dissociation between white and grey matter pathology. Patients were separated into a group with predominately white matter damage phenotype without severe signs of neurodegeneration showing high T2-LL and high NBV (WMD) and into a predominately grey matter damage phenotype having low T2-LL and low NBV (GMD). An overview of the stratification procedure is shown in Fig. 1.

### Analyses of rfMRI data

In the first step, individual resting state data were preprocessed using FEAT (FMRIB’s Expert Analysis Tool, v 6.0, part of FSL v 5.0.4). Individual pre-statistical processing included: motion correction using MCFLIRT, brain extraction, spatial smoothing using a Gaussian kernel of FWHM (full width at half maximum) of 6 mm^22^, high pass temporal filtering using a cut-off of 150 s (0.007 Hz), linear registration to the high resolution T1 scans (BBR) and non-linear registration using a warp resolution of 10 mm.

Next, Independent Component Analysis (ICA) was used for rfMRI data exploration (FSL-MELODIC, v 3.12), denoising the data and filtering out components based on high vs. low frequency content (fsl_regfilt command line tool). The resulting denoised functional images were resampled to standard space (MNI152 template 2 mm). To objectify identification of networks, we performed dual-regression analyses against ten resting-state templates from 36 healthy controls^23^ on the denoised, registered functional images of each subject in order to obtain objective individual spatial maps of the sensorimotor network (SMN).

Given the high prevalence and relevance of sensorimotor impairments in patients with MS, and importance of behavioural correlates (EDSS) for interpretation of rfMRI findings, we specifically focused on the SMN^24^. Hence, group functional connectivity maps of the SMN were computed for all groups and assessed for statistical significance (using “FSL Randomise”).

### Regions of interest

**Figure 2:**
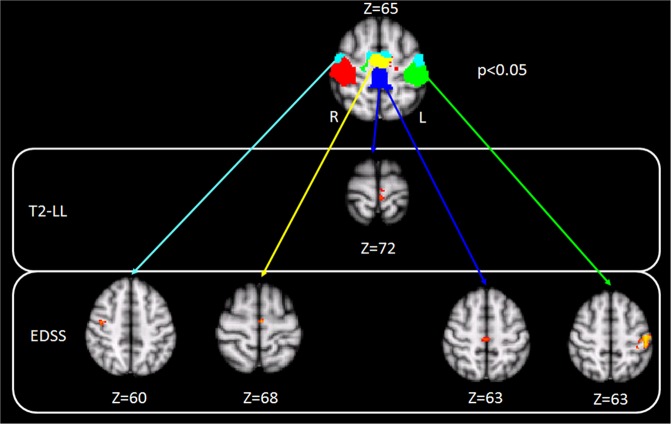
Correlation of EDSS and T2-LL with FC in the SMN. Higher EDSS scores and higher T2-LL correlate with lower functional connectivity in SMN sub-units in MS patients with a primarily white matter damage MRI phenotype.

Given the fact that functional reorganization mechanisms might differ between primary and supplementary motor areas, we further used five pre-defined functional sub-units of the SMN identified by Instantaneous Connectivity Parcellation (ICP) to assess possible differences and relationships of FC in a more refined approach^25^. These sub-units reflect biologically valid subdivisions of the SMN that adhere to known cytoarchitectural features^25^. We included ICP number 21 comprising the anterior SMN bilateral (shown in light blue, Fig. 2), ICP 22 (overlapping the supplementary motor area, shown in yellow Fig. 2), ICP 25 (overlapping the left hand area shown in green, Fig. 2), ICP 26 (overlapping the right hand area shown in red, Fig. 2) and a central SMN sub-unit, ICP 27 (overlapping the leg area, shown in dark blue, Fig. 2).

Sex- and age-corrected group differences of FC within the SMN and five sub-units of the SMN were assessed (using FSL Randomise, GLM with additional covariates).

Furthermore, sex- and age-corrected correlations between FC within each sub-network and morphological (NBV, T2-LL), as well as clinical (EDSS) measures, were computed for each group separately.

### General statistical analysis

Clinical and morphological scores were analyzed with the Statistical Package of Social Science (IBM SPSS Statistics 23). The level of significance was set at 0.05. Comparisons concerning morphological (NBV, T2-LL) and clinical variables (EDSS, disease duration) between groups were done using unpaired t-tests or ANOVA for parametric and Mann Whitney U or Kruskal-Wallis tests for non-parametric dependent variables. Partial spearman correlations were performed correcting for age.

## Results

### Clinical and morphological features of patients with predominantely white and grey matter damage MRI-phenotypes compared to healthy controls

From the entire cohort of 180 patients, 74 patients with a distinct MRI phenotype were identified and considered for subsequent analyses based on the a priori defined MRI-stratification (see Fig. 1). This algorithm led to the identification of 37 patients with a predominately white matter damage MRI-phenotype (WMD) and 37 patients with a predominately grey matter damage MRI-phenotype (GMD).

Detailed characteristics of the entire patient cohort, the two subgroups with distinct MRI phenotypes and healthy controls (HC) are presented in Table 1.

**Table 1:** Demographics, clinical and MRI data of the entire cohort of patients with MS and identified subgroups, as well as healthy controls.

	MS	WMD	GMD	HC	*p*	*p*
	180	37 (21%)	37 (21%)	37	WMD vs GMD	WMD vs GMD vs HC
sex (f)	114 (63%)	30 (81%)	20 (54%)	13 (35%)	0.024	0.001
Age, years	35.9 (9.8)	31.8 (6.9)	39.1 (9.9)	35.8 (7.9)*	0.001	0.001
EDSS	1.0 (2.1)	1.0 (1.9)	1.5 (1.8)	—	0.312	
Min-Max	0–6.0	0–4.0	0–5.5			
DD	5.0 (8.0)	5.5 (8.0)	3.5 (9.0)	—	0.969	
DMT	67 (37%)	12 (32%)	22 (59%)	—	0.062	
Clinical phenotype				—	0.472	
CIS	61 (34%)	12 (32%)	13 (35%)			
RRMS	114 (63%)	24 (65%)	24 (65%)			
SPMS	5 (3%)	1 (3%)				
NBV cm³	1516.2 (74.0)	1564.9 (36.1)	1473.6 (41.1)	1567.7 (71.8)	<0.0001	<0.0001
T2-LL cm³	16.9 (15.6)	22.6 (11.6)	7.2 (2.8)	—	<0.0001	

Nominal data (sex) is shown in number of patients (N) and percentage (%). For continuous variables (age), mean and standard deviation (SD) are presented and for non-parametric data (EDSS, DD), median and interquartile range (IQR) are shown. CIS = clinically isolated syndrome, EDSS = Expanded Disability Status Scale, DD = disease duration, DMT = patients on disease modifying treatment; f = female, GMD = predominately grey matter damage group,HC = healthy controls, NBV = normalized brain volume; T2-LL = T2-lesion load; RRMS = relapsing-remitting MS, SPMS = secondary progressive MS, WMD = predominately white matter damage group.

Patients of the WMD-group were more often female and younger compared to GMD patients. The two groups did not differ significantly regarding EDSS, disease duration (DD) and distribution of clinical phenotypes. Higher EDSS scores correlated with higher T2-LL for all 180 patients (*r* = 0.21, *p* = 0.007, age-corrected) and within the WMD group (*r* = 0.357, *p* = 0.035, age corrected). Higher EDSS scores correlated with lower NBV (*r* = −0.266, *p* = 0.001, age corrected) for all 180 patients with MS, but not within the two subgroups.

The HC group did not differ compared to both groups with distinct MRI phenotypes regarding to age, but comprised less women. WMD patients and HC had comparable NBV and both had higher brain volumes compared to GMD patients.

### Functional connectivity differences between patients with predominantely white vs. grey matter damage MRI-phenotypes compared to healthy controls

**Figure 3:**
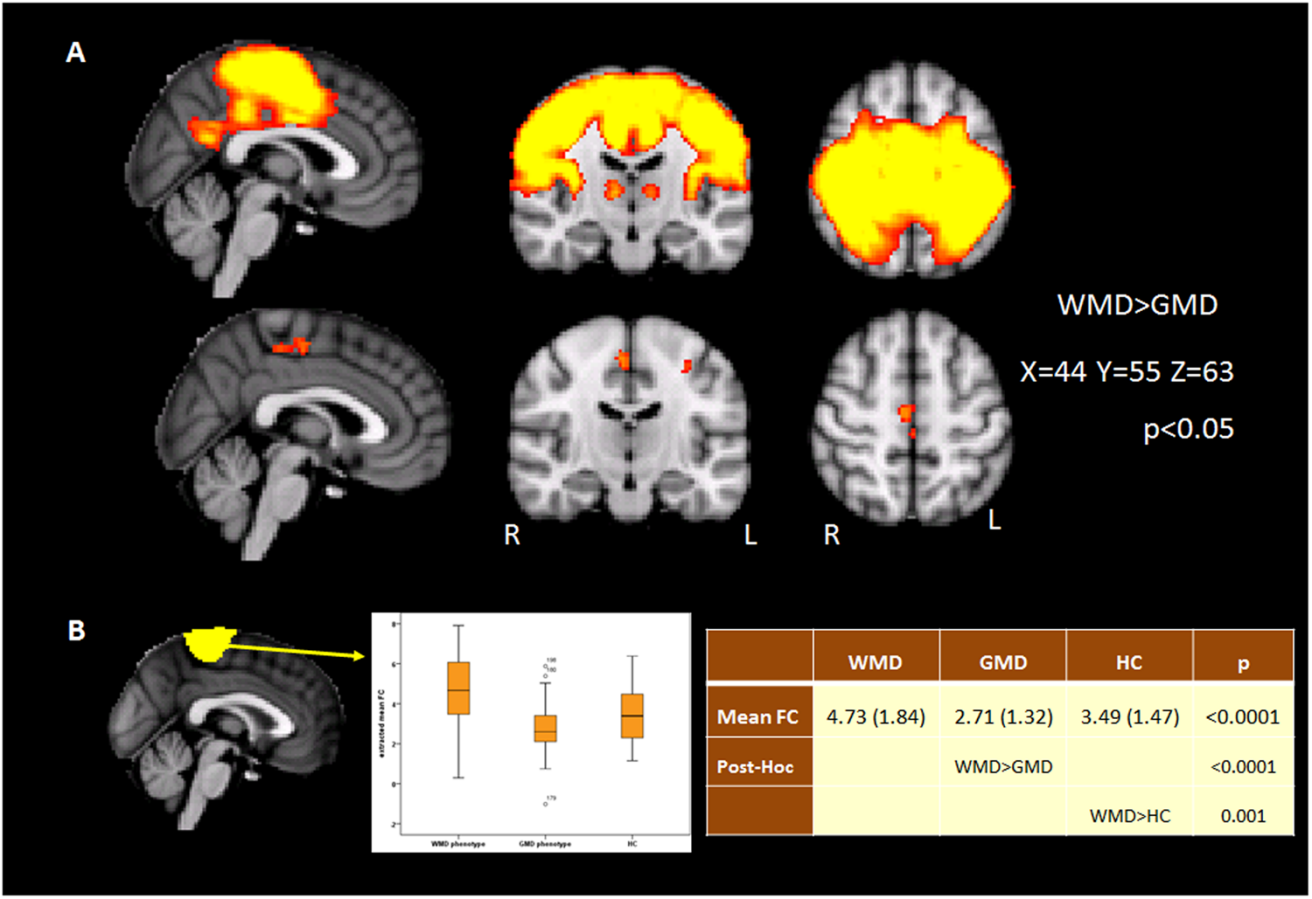
(**A**) Increased FC in the sensorimotor network in patients with the primarily white (WMD) compared to the grey matter damage (GMD) MRI-phenotype. (**B**) Increased mean FC in the central sub-unit (leg area) in WMD patients compared to GMD and HC.

WMD patients showed higher FC in the SMN in the precentral gyri bilaterally and supplementary motor area (SMA) compared to GMD patients, even after correcting for differences in sex and age (Fig. 3). No significant whole brain differences were observed compared to HC, but region of interest analysis showed that WMD patients showed increased FC compared to HC and GMD patients in the central SMN (Fig. 3).

Higher FC in all five ICP sub-units of the SMN were observed for WMD patients compared to GMD patients in the precentral gyrus and SMA.

### Correlations between functional connectivity, EDSS and morphological MRI metrics

All correlations between FC and EDSS or morphologic MRI metrics (T2-LL, NBV) were sex- and age-corrected.

Higher T2-LL was correlated with lower FC in the central SMN (leg area) in the WMD MRI-phenotype (Fig. 2; *r* = −0.357, *p* = 0.025 for extracted mean FC).

Higher EDSS scores (i.e. higher levels of physical disability) correlated with lower FC in four sub-units of the SMN (Fig. 2) in the WMD MRI-phenotype (correlation coefficients according to Fig. 2 for extracted mean FC left to right: *r* = −0.419, *p* = 0.012; *r* = −0.396, *p* = 0.019; *r* = −0.489, *p* = 0.003; *r* = −0.471, *p* = 0.004; see online Supplementary Fig. S1).

No correlations between T2-LL or EDSS scores and FC were observed for the GMD MRI-phenotype.

No correlations between NBV and FC were observed in any group.

## Discussion

We found that patients with a predominately white matter damage MRI-phenotype showed increased FC in the sensorimotor network compared to those with a predominately grey matter damage MRI-phenotype. Decreased FC was further associated with higher EDSS scores and higher T2-LL, only in the WMD MRI-phenotype. Both groups did not differ regarding EDSS or disease duration and we controlled for potential confounding influences of sex and age.

Given the complexity and heterogeneity of MS in terms of clinical manifestations, clinical course and structural abnormalities^26^, a MRI-based stratification of patients has been proposed. The clinical-MRI paradox describes the poor correlation between conventional MRI measures and clinical disability^27^. Possible explanations for this include, on the one hand, the well-known limitations of conventional MRI (such as its inability to quantify MS related damage occurring within and outside T2-visible lesions) and, on the other hand, the shortcomings of the Expanded Disability Status Scale (EDSS), which, however, still represents the most frequently used tool to quantify the severity of neurological impairment and disability in patients with MS^27^. In addition, there is increasing evidence that the severity of the clinical manifestations of MS does not simply result from the extent of tissue destruction, but it rather represents a complex balance between tissue damage, tissue repair, and functional reorganization^10^. The application of functional MRI (fMRI)^9,28,29^ helped to improve our understanding of this complex interplay.

Several rfMRI studies have suggested altered FC patterns in patients with MS compared to healthy controls reporting that this technique is sensitive to brain functional reorganization^30^. Also differences in FC between clinical phenotypes have been reported, suggesting that an a priori stratification of patients with MS might be essential to assess potential functional differences^16^. However, to our knowledge this is the only study to investigate whether FC differs between distinct phenotypes defined by MRI. Therefore, we specifically focused on the two groups with a dissociation of a predominately WMD compared to the GMD phenotype defined by MRI. A stratification based on MRI measures provides an objective classification and reflects differential pathophysiologic mechanisms of MS. A new taxonomy on the basis of mechanisms rather than clinical empiricism could improve prediction of disease course and treatment^2,31^.

At group-level, increased SMN FC in the WMD group was observed, which could reflect a compensation mechanism given comparable EDSS scores between the groups. Region of interest analysis showed increased FC in the WMD group in the central SMN (leg area) also compared to healthy controls. However, within the WMD group, higher T2-LL and higher EDSS correlated with decreasing FC, suggesting that above a certain threshold of white matter damage, functional compensation of the network capacity breaks down^32^. Although our findings seem to support this network collapse theory, further confirmation in longitudinal studies is needed to explore functional reorganization in distinct MRI-phenotypes.

Furthermore, we found decreased FC in the central SMN (leg area) to be associated with increased focal white matter changes in WMD patients. This is in line with previous studies reporting an association between increased FC and decreased structural connectivity in the SMN in patients with MS^33^. Longitudinal studies have to determine whether such functional changes confer a systematic vulnerability to disease progression or, conversely, protect against the onset of deficits^26,34^.

Some limitations of our study have to be considered when interpreting our results. First, we only assessed cross-sectional differences of SMN FC in MRI-phenotypes and HC. Although previous studies showed that rfMRI networks are stable in MS,^35^ longitudinal assessments of rfMRI are essential to assess whether increases of FC are adaptive or maladaptive. Secondly, we are aware that more refined stratifications of patients based on MRI-phenotypes exist^2^. However, it was shown that unique subpopulations can be reliably identified using a simple distinction based on T2-lesion load and brain volume measures^4^. Furthermore, we aimed to use a potentially clinically applicable stratification based on conventional MRI sequences and therefore decided to use global T2-LL and NBV for identification of distinct groups. Thirdly, the EDSS range of our sample was rather narrow, including primarily patients with mild impairment. Further studies extending these findings within a sample with a broader spectrum of disease severity are warranted. Fourth, given the importance of behavioural correlates (e.g. EDSS) for interpretation of rfMRI findings we did not assess potential differences of other networks (e.g. cognitive networks or the default-mode network).

Also more refined quantitative MRI measures (such as e.g. magnetization transfer imaging^36,37^) might be useful to better understand the relationship between (micro)structural changes and functional reorganization in MRI-phenotypes. Furthermore, an additional consideration of e.g. the T1 black hole ratio might help to improve such stratification, however for this first approach we focused on T2-LL as a cumulative measure of the footprints of focal inflammation.

In summary, our findings suggest that mechanisms of functional reorganization differ between distinct phenotypes defined by MRI and therefore, such a stratification might be useful when A) assessing functional reorganization in large MS cohorts to get a clearer picture of the structure-function relationship in such MRI phenotypes or B) selecting patients for e.g. rehabilitative interventions as functional compensation mechanisms might differ between MRI phenotypes.

## Supplementary information

Supplementary Information

## Data Availability

The data that support the findings of this study are available from the corresponding author upon reasonable request.
