# Supplementary material for: Morphological MRI phenotypes of multiple sclerosis differ in resting-state brain function
Source: Sci Rep. 2019 Nov 7;9:16221. doi: 10.1038/s41598-019-52757-7 (PMC6838050; doi:10.1038/s41598-019-52757-7)

**Morphological MRI phenotypes of multiple sclerosis differ in resting-state brain function**

Daniela Pinter (PhD), Christian F. Beckmann (PhD), Franz Fazekas (MD), Michael Khalil (MD, PhD), Alexander Pichler (MD, PhD), Thomas Gattringer (MD, PhD), Stefan Ropele (PhD), Siegrid Fuchs (MD), Christian Enzinger (MD)

**Supplementary Figure S1:** Correlation plots of higher EDSS scores with lower extracted mean functional connectivity (FC) within the WMD group.


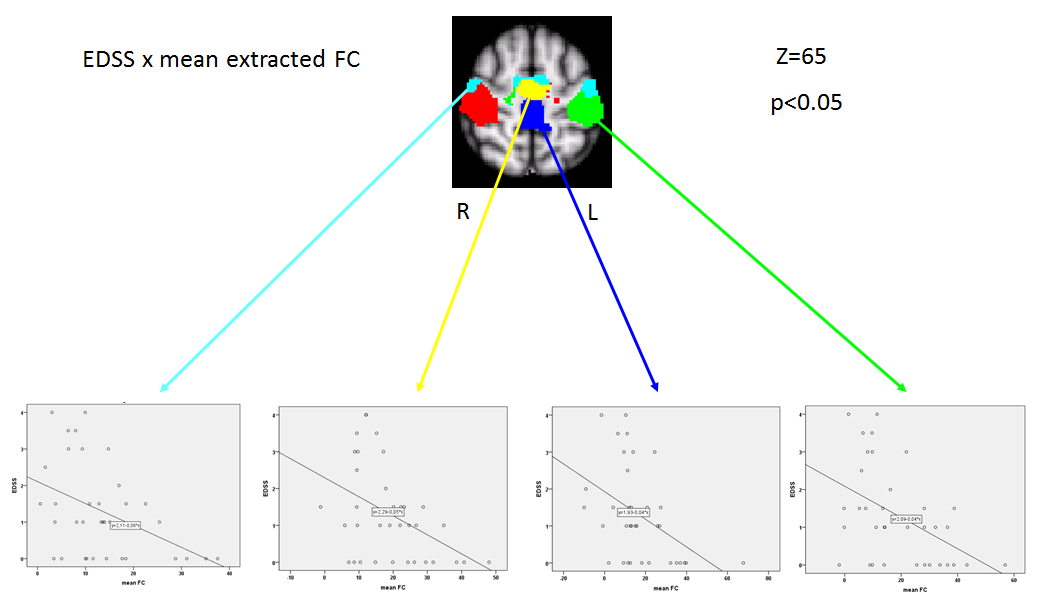

Supplement: Supplementary file 1 — Supplementary Information [file 41598_2019_52757_MOESM1_ESM.docx]
